# The risks of unconcern: low sensitivity to threat can have unfortunate consequences

**DOI:** 10.3389/fpsyg.2024.1390968

**Published:** 2024-11-13

**Authors:** Stephen L. Ristvedt

**Affiliations:** Department of Anesthesiology, Washington University School of Medicine, St. Louis, MO, United States

**Keywords:** trait anxiety, sex differences, threat sensitivity, defensive survival circuits, risk perception, personality theory, health psychology

## Abstract

Each one of us is confronted with warnings of danger or threats to wellbeing in our everyday life, whether in the form of certain road signs, Public Service Announcements, ominous changes in bodily functioning, or cautionary tales heard from family or friends. There is great inter-individual variation in how people respond to such threats, with some people habitually tending to ignore or dismiss them, often to their peril. The first purpose of the present paper is to review several studies showing that individuals—most often men—who score very low on measures of trait anxiety are more likely to engage in behaviors that could jeopardize their physical wellbeing. The general hypothesis that is derived from that review is that when attention to everyday threats is chronically muted by way of a dispositional trait, the likelihood of proceeding down some dangerous path is increased. Those findings are then discussed within the broader context of personality theory to highlight the importance of recognizing the bipolarity of common traits. Here the case is made for replacing the term trait anxiety with the term threat sensitivity in order to capture the full breadth of this basic personality variable. A discussion of the neurobiological underpinnings of threat sensitivity is then presented with an emphasis on individual and sex differences in the workings of the defensive survival circuitry. Taken together, this paper has implications for two subfields within psychology. For the area of personality theory, this paper provides support for the adaptationist view with the argument that low threat sensitivity has both adaptive and maladaptive potential. For the area of health psychology, it is argued that some individuals who demonstrate a habitual tendency to neglect their physical wellbeing may be acting—at least in part—in accordance with their innate neurobiological constitution.

“I do not in the least mind risk to my life.”—Theodore Roosevelt (1913)

## Introduction

Those words were penned by Roosevelt in a letter to his long-time friend Father John Zahm during the early planning stages of his legendary expedition through the Brazilian jungles down the perilous River of Doubt (Roosevelt, 1914). It turned out to be an ill-conceived venture that would kill three of Roosevelt’s fellow travelers and that would very nearly kill Roosevelt himself, whether by severe malnutrition, exhaustion or malaria infection, all of which he suffered, or by suicide, which he threatened. So, he had indeed put his life at risk, and he never fully recovered from the physical toll of that trip before he died less than 5 years later (Millard, 2005). If one were to describe Roosevelt’s personality by drawing from a lexicon of trait adjectives, with knowledge of this and his other well-documented exploits, terms like “fearless” and “bold” would clearly top the list. But when the outcome of this particular expedition is also known, one might add terms like “careless” or “imprudent.” These four traits would seem to cluster together in certain individuals—not just Roosevelt—who possess a dispositional tendency to put or find themselves in situations whose threatening aspects are downplayed or ignored. Pertinent to the present topic, this phenomenon also appears on a much more mundane scale in which some people seem prone to overlook everyday threats. Not surprisingly, trouble often ensues.

The purpose of this paper is to contribute to an examination of the psychological makeup of these individuals who are, more often than not, men. This academic exploration will proceed as follows. First, I will review a disparate collection of studies that all demonstrate associations between an exceptionally low level of trait anxiety, as operationalized in a number of ways, and various behavioral manifestations of neglect of personal wellbeing. Given the correlational design of some of these studies, the important issue of causality will be addressed in depth. Second, I will discuss how a reconsideration of terminology—from “trait anxiety” to “threat sensitivity”—allows a fuller understanding of the continuous and bipolar nature of threat sensitivity as a very basic personality variable. Viewing the full continuum of this variable also helps to shed light on the fact that some sort of maladaptation can occur at both tails of the continuum. Third, I will undertake an examination of the neurobiological underpinnings of threat sensitivity, which is included here to support the hypothesis that individual differences in the neglect of physical wellbeing may have innate constitutional roots. Lastly, the Discussion section includes some speculation regarding the adaptive potential of low threat sensitivity. That is, individuals who tend not to be deterred by threats that may stop others are then freer to pursue the expansion of their personal, societal, and geographic footprints.

## Empirical studies

### Mountain climbers

Theodore Roosevelt was but one example of individuals who embark on extreme adventures. Each year, for example, hundreds of mountaineers attempt to reach the summit of Mount Everest, even though the odds of these adventurers completing their trips unscathed are clearly not favorable; one in a hundred of them die in the attempt (Huey et al., 2020). Unlike with Roosevelt, however, researchers have been able to assess aspects of the psychological profiles of these modern-day explorers with self-report measures that have been developed to operationalize current theories of personality. For example, the Eysenck Personality Questionnaire—Revised (EPQ-R; Eysenck and Eysenck, 1991) was given to 39 climbers during their 2 weeks acclimatization phase at the Mount Everest base camp prior to their ascent (Egan and Stelmack, 2003). Compared to a sample of 31–40 year-old males who had provided standardization data for the development of the EPQ-R, these climbers had lower scores on the Neuroticism scale and higher scores on the Psychoticism/Tough-mindedness and Extraversion scales. In other words, the climbers were less prone to anxiety and worry and tended more toward aggressiveness, dominance, boldness, and risk-taking. In a similar study, 41 Mount Everest climbers completed several questionnaires prior to their ascent that included an abbreviated version of the BIS-BAS scales (Carver and White, 1994; Carver et al., 2000) as well as investigator-prepared measures of “state anxiety” and “excitement,” assessing how they were feeling “right now” (Feldman et al., 2013). In this study, the climbers were queried again upon their return to the base camp to report on the relative success of their climb. Scores on the pre-climb anxiety scale were inversely related with the altitude that was attained and were lower for those who had reached the summit compared to those who had not. It is important to note that, even though the anxiety measure assessed how participants were feeling “right now,” the climbers’ scores were associated with their performance 3–4 days later, which brings into question whether the motivational aspect of that “feeling” was really only transitory. Despite that finding, however, there were no significant associations between any of the performance measures and scores on the BIS (Carver and White, 1994), which is generally considered to be a measure of trait anxiety. Lastly, those who had reached the summit had higher scores on the BIS-BAS Reward Responsiveness scale than those who did not. That scale was developed to measure positive affective responses to the occurrence or anticipation of reward (e.g., “When I see an opportunity for something I like, I get excited right away”).

These examples of extreme adventurers are particularly spectacular illustrations of people who appear to be temperamentally dismissive of seemingly obvious threats and who are thus more willing to put themselves in situations where the risks are high. Note that both of these small studies provide some evidence for two basic components in the psychological makeup of these individuals. The first component in the average profile is a relative lack of anxiety or fear, which will be the focus of the remainder of this paper. The second component, which could be characterized as a tendency toward boldness and a motivation to seek reward, will be considered much more briefly later in this paper along with a discussion of how these two components may interact.

The finding that these mountain climbers reported relatively low levels of anxiety is certainly no surprise on the face of it. How else could one voluntarily undertake a complex challenge that carries with it a sizeable degree of risk to physical wellbeing and even survival? But while the threats faced by those adventurers are obvious and extreme, each one of us is confronted with much more ordinary threats or warnings of danger in our everyday lives, whether in the form of certain road signs, Public Service Announcements, ominous changes in bodily functioning, or cautionary tales heard from family or friends. Clearly, there are some people who tend to disregard such warnings. A collection of studies, to which our attention now turns, suggests that a variety of forms of trouble can ensue when people who score very low on measures of trait anxiety dismiss more common threats to their wellbeing.

### Neglect of serious physical symptoms

A fairly common situation in which a threat to one’s health and wellbeing may be overlooked is when a sign or symptom of some serious disease begins to emerge. When proper detection, diagnosis and treatment are delayed, the chances of an unfortunate outcome are increased. In the case of cancer, for example, even a 4 weeks delay in treatment is associated with increased mortality (Hanna et al., 2020). We first investigated this phenomenon in a study of patients who had recently been diagnosed with rectal cancer in an attempt to understand the individual characteristics associated with the amount of time that it took them to seek and obtain appropriate help (Ristvedt and Trinkaus, 2005). Rectal cancer was chosen for this study because of several aspects of the disease that would facilitate a study of delays in medical consultation. First, because of their distal location in the gastrointestinal tract, tumors in the rectum are usually first signaled by fresh red blood in the stool, which provides a visual stimulus that should be innately threatening. Second, however, blood in the stool could easily be attributed to either serious (cancer) or benign (hemorrhoids) causes, which leaves much room for individual differences in interpretation and behavioral response. Third, the salience of that sign increases gradually over an extended period of weeks and months, which allows for wide variability in the amount of time that it takes for the afflicted person to respond. And lastly, the perception of such signs of rectal cancer is a very private matter, so that the initial response is not at all dependent on the influence or advice of others.

The participants in this study were 69 patients who had recently been diagnosed with rectal cancer. They completed a paper-and-pencil questionnaire that had been developed in pilot work to collect information about the history of their symptoms and their pursuit of medical care. Similar to previous research (e.g., Andersen et al., 1995), three pivotal events along the trajectory from symptom onset to medical consultation were identified: (1) the point at which signs or symptoms were first noticed by the patient, (2) the point at which the person decided that those bodily changes might be signaling some serious health problem, and (3) the point at which the person first saw or called a doctor about the problem. Participants were asked to estimate how much time (in weeks) had elapsed between points (1) and (2) (“Symptom Appraisal” time) and between points (2) and (3) (“Action Appraisal” time). This method allows investigation of psychosocial variables that might correlate with the lengths of these two sequential stages in an effort to better understand the possible causes of delays in seeking medical care (Safer et al., 1979; Cacioppo et al., 1986; Andersen et al., 1995; Weller et al., 2012). It was found that, on average, symptom appraisal time accounted for the majority (about 70%) of the total time prior to medical consultation, which is similar to other studies of delay in cancer (Cacioppo et al., 1986; Andersen et al., 1995). Furthermore, 16 patients took 6 months or more and eight patients took 1 year or more to realize that their symptoms might be signaling some serious health problem. These findings point to the importance of examining individual differences in the perception of possibly threatening stimuli related to health.

To that end, analyses were conducted to determine the association between the length of symptom appraisal time and scores on the Harm Avoidance scale of the Temperament and Character Inventory (TCI-HA; Cloninger et al., 1994). Based on findings from pilot work, TCI-HA scores were divided into tertiles—low, medium, and high—and entered into time-to-event analyses along with age at diagnosis, sex, and education level predicting length of Symptom Appraisal time. Single variable Kaplan–Meier estimates of median symptom appraisal times showed that patients in the lowest TCI-HA tertile took significantly longer (30.0 weeks) than their middle (9.0 weeks) and high (12.0 weeks) tertile counterparts to recognize the seriousness of their symptoms. All of these analyses were repeated using the Trait scale of the State–Trait Anxiety Inventory (STAI-T; Spielberger et al., 1970), although the global null hypothesis was not rejected. Parenthetically, patients with lower educational levels (high school or less) had longer median Symptom Appraisal times (15.0 weeks) than patients with higher educational levels (8.0 weeks), but there were no significant associations between symptom appraisal times and either sex or age at diagnosis.

**Figure 1:**
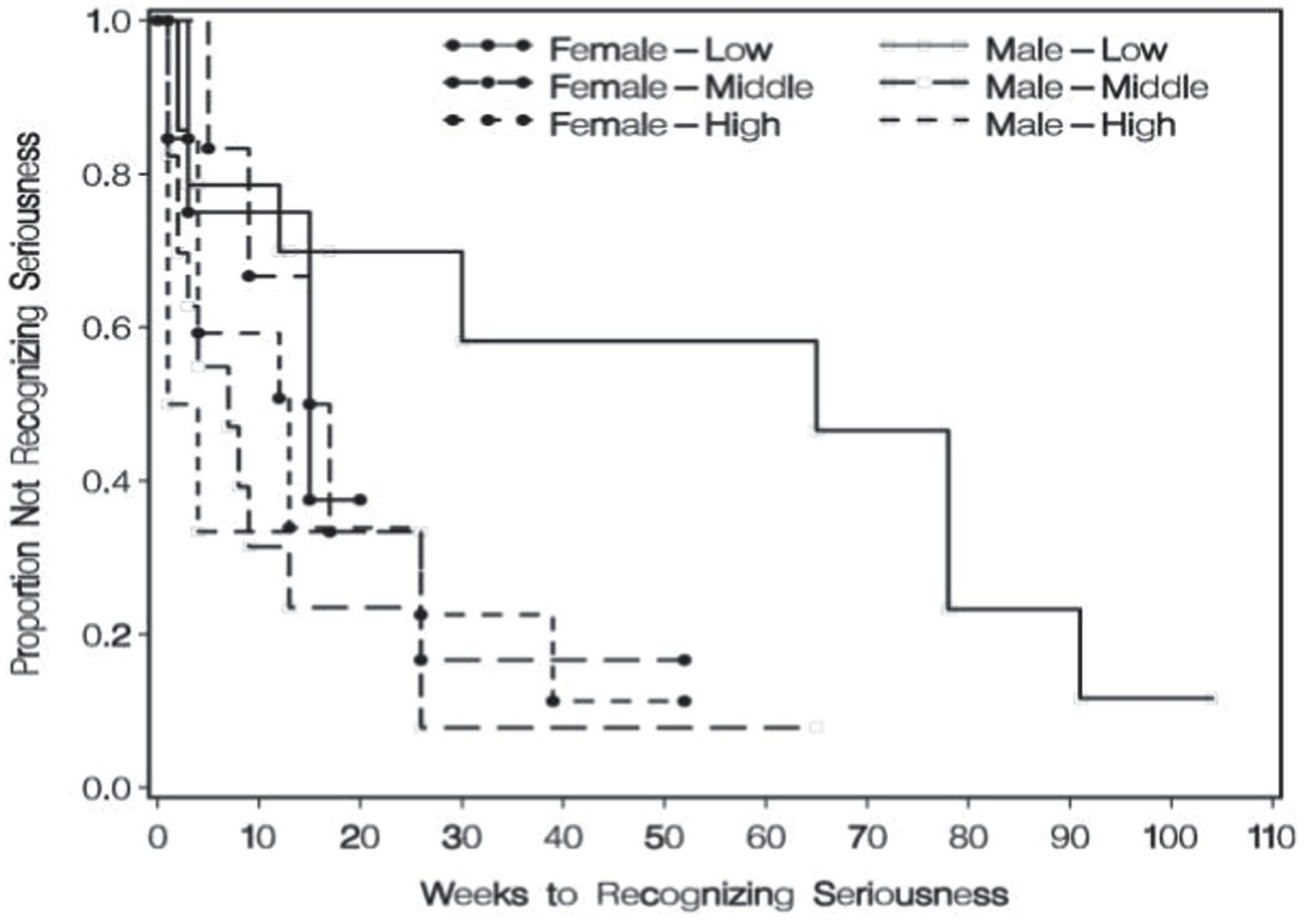
Symptom appraisal time by sex and TCI-HA tertile. From “Sex differences in responding to rectal cancer symptoms,” by Ristvedt and Trinkaus (2008).Copyright 2008 by Taylor & Francis. Reprinted with permission.

Shortly after those findings were published, further examination of the extant literature uncovered a handful of articles suggesting that there may be some utility in considering the interaction between sex and negative emotional functioning in the reporting of physical symptoms (Gijsbers van Wijk and Kolk, 1997; Williams and Wiebe, 2000; Van Diest et al., 2005). We then undertook to analyze the same data described above, but this time including the interaction between the sex of the patient and TCI-HA scores in the prediction of symptom appraisal time. A Cox proportional hazards time-to-event analysis of symptom appraisal time was run with the covariates being sex, TCI-HA score, and the sex by TCI-HA score interaction. The global null hypothesis was rejected. Although neither sex nor TCI-HA score were independent predictors in this model, the interaction between the two did reach statistical significance. The pattern of findings is clearly seen in Figure 1, which illustrates Kaplan–Meier curves for each of the six subgroups. Each downturn in a line represents another person or group of persons reaching the event of interest, i.e., the point at which they recognized the seriousness of their rectal cancer symptoms. As can be seen, males who had the lowest TCI-HA scores tended to take much longer than members of the other five subgroups (Ristvedt and Trinkaus, 2008).

Those findings encouraged us to expand our investigation into the interaction between sex and trait anxiety in the association with the duration of the symptom appraisal stage, but this time with a larger sample of patients, with a broader range of colon and rectal cancers (and thus symptom types), and using a different measure of trait anxiety, the Behavioral Inhibition Scale (BIS; Carver and White, 1994). A Cox proportional hazards model again found a significant interaction between sex of the patient and their BIS scores, split at the median. Males with low BIS scores took significantly longer than their high BIS score male counterparts to recognize the seriousness of their symptoms (medians of 17.0 vs. 2.0 weeks). This finding provided an independent replication of the earlier finding (Ristvedt and Trinkaus, 2008). Parenthetically, in an interesting and unexpected twist, females with *high* BIS scores took significantly longer than their *low* BIS score female counterparts to recognize the seriousness of their symptoms (medians of 26.0v vs. 9.0 weeks). Although this second finding is outside the scope of the present paper, there was some evidence in that report that the high BIS females were more likely to attribute their symptoms, which are much more vague in colon cancers, to stress (Ristvedt et al., 2014). Further study may clarify that interpretation.

### Healthcare utilization

With those findings in mind, another study was conducted to test whether males who are low in trait anxiety might also stand apart from their higher anxiety counterparts in seeking medical care more generally. That question was addressed as part of a community-based study of healthcare utilization among a sample of 483 African American men living in a large Midwestern urban area. Participants were asked to identify their typical source of healthcare as well as how often they sought medical consultation or assistance. They also completed a large battery of psychosocial measures, all of which were hypothesized to have some association with healthcare utilization. Included among those measures was the BIS (Carver and White, 1994). Ordinary logistic regression analyses were used to compare three subgroups of men according to their usual source of healthcare: a doctor’s office or clinic, an emergency room, or no place. The three variables that reached statistical significance in differentiating men by their usual source of healthcare were age, insurance status, and BIS scores. With regard to the BIS scores (and after adjusting for age and insurance status), men who had no usual source of healthcare had lower BIS scores than men who utilized a doctor’s office or clinic, who in turn had lower BIS scores than those men who tended to use an emergency room. In other words, those men with the lowest levels of trait anxiety were the ones who were least likely to identify any specific healthcare source that they routinely used for consultation or care (Ristvedt et al., 2019).

### Accidents

Individuals who demonstrate a tendency to overlook threats to their physical wellbeing may also be more likely to be involved and possibly injured in accidents. The data for this next analysis were taken from a large longitudinal study that showed a relationship between low anxiety and involvement in accidents—both fatal and nonfatal (Lee et al., 2006). Participants in that study were drawn from the cohort of all individuals who were born in England, Wales, or Scotland in the week of March 3–9, 1946 and who had been selected to take part in the Medical Research Council National Survey of Health and Development (MRC NSHD) study. The original sample consisted of 5,362 individuals stratified by social class. Beginning when participants were infants until the present, assessments of development and individual functioning have been provided every few years by parents, teachers, health visitors, and eventually, by the participants themselves.

Trait anxiety was measured by means of assessments obtained as part of the broad NSHD study. When participants were 13 years old, their teachers were asked, *“Would you describe this child as an anxious child*—i.e. *apprehensive, worried and fearful?”* Teachers were told to choose from *“not at all,” “somewhat,”* or *“very”* in their responses. Because very few participants were judged to be in the “very” anxious group, participants were categorized into either Low (“not at all”) or High (both “somewhat” and “very”) trait anxiety groups. Assessment of involvement in fatal accidents was captured using mortality data derived from the U.K.’s National Health Service Registry. Assessment of involvement in nonfatal accidents was derived from self-report measures that were included in the NSHD data collections that occurred when participants were 24 years old. Participants were asked to report: (1) any accidents in which they were involved since the time of the previous data collection, and (2) whether any medical treatment had been sought as a result of any reported accidents. Participants’ answers to these two questions constituted the measure of involvement in nonfatal accidents that required medical treatment.

Two secondary analyses of the data from that original study were conducted in order to model the interactive effect of trait anxiety-by-sex on involvement in accidents (Lee and Ristvedt, 2010). The first of those analyses concerned the number of accidental deaths that occurred between the ages of 13 and 25 years among the 4,070 participants who had been assessed for trait anxiety at 13 years of age. The left-hand side of Table 1 shows the numbers of participants who were assessed as having a low or high anxiety level, as categorized for male, female, or both sexes combined. The right-hand side of the table shows the numbers of participants who died by accident by age 25, again categorized by trait anxiety level and by sex. As may be observed, a total of 18 of the original participants (males and females combined) had died by accident by age 25, and males (*n* = 16) far outnumbered females (*n* = 2) in the number of accidental deaths. Also, 15 of the 16 males who died by accident had been judged to be low in trait anxiety as compared to only 1 male who had been judged to be high in trait anxiety. One might infer that the 15 low anxious males who died by accident had been more likely than their three unfortunate peers to put themselves into perilous situations, perhaps by ignoring the inherent threats. Cox regression analyses indicate that low trait anxiety was a significant risk factor for death by accident among males (HR = 10.5; *p* < 0.05), but not among females (HR = 1.0; n.s.).

**Table 1:** Accidental deaths prior to age 25, by sex and trait anxiety.

Trait anxiety				N deaths (rate × 10^−4^)		
	N assessed (Person years at risk)			HR (95% CI)		
	Both sexes	Males	Females	Both sexes	Males	Females
Low	2,222 (24,342)	1,249 (13,663)	973 (10,679)	16 (6.57)	15 (11.0)	1 (0.94)
High	1,848 (20,300)	870 (9,548)	978 (10,752)	2 (0.99)	1 (1.05)	1 (0.93)
				*5.9 (1.3–25.5)	*10.5 (1.4–79.3)	1.0 (0.1–16.1)

*p < 0.05; HR > 1 indicates that the low anxiety group had greater mortality than the high anxiety group.

Ordinal regression was used in the second analysis to model the interactive effect of trait anxiety-by-sex on the involvement in nonfatal accidents. Of the 4,070 participants who had been assessed for trait anxiety at age 13, a subset of 3,987 of them had also provided information periodically between the ages of 13 and 25 regarding the occurrence of nonfatal accidents that prompted the seeking of hospital treatment. Table 2 shows the data regarding the number of self-reported hospital-treated accidents that occurred up until the age of 25 years. Again, the left-hand side of the table shows the numbers of participants who were assessed as having low or high anxiety, for the entire cohort as well as for males and females separately. The right-hand side of the table shows the numbers of hospital-treated accidents that had been reported by this group between the ages of 13 and 25, again categorized by trait anxiety level and by sex. As can be seen, low anxiety males suffered a significantly higher accident rate of 11.62 × 10^−2^, compared to a rate of 10.31 × 10^−2^ for high anxiety males (OR = 1.2; *p* < 0.05). But there was no significant difference in accident rates between females who were low in anxiety (4.66 × 10^−2^) and females who were high in anxiety (4.39 × 10^−2^).

**Table 2:** Self-reported hospital-treated accidents prior to age 25, by sex and trait anxiety.

Trait anxiety				N accidents (rate × 10^−2^)		
	N assessed (Person years at risk)			OR (95% CI)		
	Both sexes	Males	Females	Both sexes	Males	Females
Low	2,177 (23,872)	1,220 (13,360)	957 (10,512)	2,043 (8.56)	1,553 (11.62)	490 (4.66)
High	1,810 (19,900)	854 (9,388)	956 (10,512)	1,430 (7.19)	968 (10.31)	462 (4.39)
				*1.1 (1.0–1.3)	*1.2 (1.0–1.4)	1.0 (0.9–1.3)

*p < 0.05; OR > 1 indicates that the low anxiety group had more self-reported hospital-treated accidents than the high anxiety group.

## Summary of empirical studies: limitations and strengths

### Limitations

The studies reviewed above all converge on the same general pattern showing that low anxiety males are more likely than their peers to find or put themselves in potentially perilous circumstances. At the same time, however, all of those studies have weaknesses that call that interpretation into question. First, the studies of mountain climbers were based on very small samples, albeit for obvious reasons. The remaining studies, although based on respectable sample sizes, had limitations of their own. In the healthcare utilization study, all participants were African American males, which did not allow comparisons to be made with other racial/ethnic groups nor with females. However, the relationship between participants’ levels of trait anxiety and their usual source of healthcare did still suggest that those men with lower levels of trait anxiety were more lax about their healthcare than their higher anxiety male counterparts. In the large epidemiological study of accidents, there was a very small proportion of accidental deaths, although males who were judged to be low in anxiety accounted for 15 of those 18 deaths. Also, the judgments of anxiety level in that study were based not on the students’ subjective self-reports but rather on their teachers’ objective observations. But what exactly did the teachers observe in order to reach their conclusions? One might imagine that they noticed which students displayed outward signs of trepidation when faced with potentially threatening situations and which students did not. If that was the case, then those behavioral characteristics and the underlying motivations likely persisted beyond the time of the initial teachers’ assessments.

The most obvious limitation to any of those studies is found in both of the symptom appraisal studies and in the healthcare utilization study, in which we claimed to have “predicted” past health-related behaviors from current measures of trait anxiety; such a claim begs further discussion and explanation (Grosz et al., 2020). In order to defend those methods and our interpretations of the results, two issues need to be addressed. First, there is the issue of *temporal* order. For our claims to hold water, we would need to argue that the psychological characteristics that were assessed by the trait anxiety measures were very similar to the psychological characteristics that could have been assessed before and at the same time that the behaviors in question occurred. In that respect, there is ample evidence to support the contention that the characteristics that are operationalized by both the TCI-HA and the BIS measures are generally stable through time.

The TCI-HA scale, for example, was developed as part of the Temperament and Character Inventory (TCI; Cloninger et al., 1994) to assess “a heritable tendency to respond intensely to aversive stimuli,” which is one aspect of Cloninger’s unified biosocial theory of personality (Cloninger, 1986, p. 167). Since its inception, hundreds of studies have been done with the TCI-HA that support its use as a measure of an enduring personality characteristic commonly termed trait anxiety (Markett et al., 2016). With respect to the temporal stability of that measure, test–retest correlations over weeks to a few years are about 0.8 in general population samples and over 0.7 in clinical samples (C. R. Cloninger, personal communication, June 8, 2023). Further support for the stability of the TCI-HA measure can be found in studies showing significant associations between scores on the measure and individual differences in genetic variation (Montag et al., 2010; Zwir et al., 2020), brain neuroanatomy (Mincic, 2015), and functional connectivity in the brain (Markett et al., 2013).

The BIS scale, on the other hand, was developed as one part of the BIS-BAS scales (Carver and White, 1994) to assess individual differences in the activity of the neurologically-based Behavioral Inhibition System, which was described by Jeffrey Gray in his original Reinforcement Sensitivity Theory (RST; Gray, 1982, 1987). According to RST, the Behavioral Inhibition System is a motivational system that inhibits any behavior that may lead to aversive outcomes and also controls feelings of anxiety and fear in response to threatening cues. Like the TCI-HA, the BIS scale has also demonstrated a high degree of temporal stability, with one study showing a test–retest correlation of 0.63 over a period of 2–3 years (Takahashi et al., 2007). Data from that same study showed that genetic factors accounted for around one-third of the variance in BIS scores (Takahashi et al., 2007); other studies have found significant associations between BIS scores and specific genetic polymorphisms (Whisman et al., 2011; Johnson et al., 2016).

Even if it is true that the psychological characteristics that influenced the participants’ responses to these two self-report measures were extant at the time that these important health-related decisions were being made, there remains the second issue of *causal* order, which is also difficult to infer given the correlational design of these studies. Did our participants’ behaviors regarding health and wellbeing influence their levels of trait anxiety or was it the other way around? Even though an intuitive answer to that question might come easily to mind, an attempt at rational argument should still be made. In his book, “The Logic of Causal Order,” Davis (1985) provided a commonsense basis for inferring the direction of the causal arrow that might exist between two contemporaneous variables that demonstrate significant correlation. He asserted that the arrow typically points from the variable that is relatively stable and difficult to change to the variable that is less stable and more subject to change. With respect to the studies reviewed above, that line of reasoning would suggest that the participants’ levels of trait anxiety likely had some influence on the healthcare decisions and behaviors that were assessed in those respective studies.

While on the subject of causality, some clarification should be offered regarding the use of the term “cause” in this context. John Stuart Mill, in his classic treatise on “A System of Logic” (Mill, 1882), noted that the “real cause” of any consequent effect typically consists of some combination of one or more relatively stable “conditions,” which may have preexisted for some indefinite period of time, along with one or more transitory antecedent “events.” With respect to the studies reviewed above, the term “conditions” would be applied to the participants’ levels of trait anxiety and their sex. The antecedent “events,” on the other hand, would be such occurrences as an opportunity to join a Mount Everest expedition or a tantalizing dare from a peer to engage in some risky—even foolhardy—behavior. Put simply, the stage is set, the cue is given, and the actor acts.

That paradigm is somewhat altered in the studies of symptom appraisal and healthcare utilization. Specifically, the combination of the conditions of low trait anxiety and male sex would function as a composite “counteracting cause” (Mill, 1882) that either slows or prevents the most prudent health-related course of action when participants were exposed to pertinent cues or events. All of the participants in the symptom appraisal studies, for example, were regularly exposed to events (e.g., rectal bleeding) that were signaling the development and progression of rectal cancer. For several weeks and even months, the low anxiety males did *not* respond to those recurrent warning signs that could have prompted more adaptive decisions and behaviors. Similarly, in the healthcare utilization study, all of the participants were probably exposed at some time or other to Public Service Announcements or social norms encouraging the establishment of a usual source of healthcare. Similar to the symptom appraisal studies, the condition of low anxiety acted as an impediment to establishing a more prudent ongoing plan for health maintenance and care.

### Strengths

Despite the limitations listed above, the strength of the studies reviewed is best revealed when they are considered altogether. That is, even though widely disparate populations were assessed and very different outcomes were measured, the findings from these studies all converge on the same general pattern: low anxiety males, whether compared to peers of both sexes or only to higher anxiety males, were more likely to put or find themselves in situations that could have led to (or did lead to) very unfortunate consequences. The collection taken together thus provides a “conceptual replication” (Diener et al., 2022) of a relationship in which males who tend to be low in anxiety also tend to display a general neglect of, or inattention to, situations that present a threat to their own physical wellbeing, which can then put that wellbeing at risk.

## Terminology and the broader continuum: threat sensitivity

On its face, it would seem that scoring extremely low on a scale of trait anxiety would be the most enviable way to live one’s life; that is, in a default state of “extreme calmness” (Siddaway et al., 2018). But the studies listed above tell a different story. Under certain circumstances, having too little anxiety in the face of a potential threat can lead to negative consequences or even disaster. In that respect, contrary to previous thinking, several authors (Pettersson et al., 2014; Mulder et al., 2016; Rojas et al., 2019; Widiger and Crego, 2019) have pointed out that there are maladaptive trait characteristics that lie at *both* extremes of all five domains of the Five Factor Model (FFM; Costa and McCrae, 2017) as well as all five domains of their theoretical offspring: the Section III personality trait system of DSM-5 (American Psychiatric Association (APA), 2013) and the proposed changes to the Mental and Behavioural Disorders section of ICD-11 (International Advisory Group for the Revision of ICD-10 Mental and Behavioural Disorders, 2011). This reconceptualization is also congruent with a dimensional as opposed to a categorical view of personality traits (Monaghan and Bizumic, 2023), which means that the most adaptive place for a person to reside, psychologically speaking, is somewhere toward the middle of these personality trait distributions (Grant and Schwartz, 2011). Hence, in line with this newer paradigm, the present paper suggests that having a modest dose of anxiety along with a ready eye toward what could go wrong can be a very good thing.

However, there is an issue of terminology here that impedes a broader understanding of the role of this particular personality trait in the full range of individual differences in motivational and behavioral tendencies related to threat. To say that a person is very low in trait anxiety is analogous to saying that a person who is 6 feet tall and weighs 120 pounds is very low in obesity. Instead, we would say that person has a very low body mass index (BMI), which is a term that covers the entire continuum from skeletally gaunt to morbidly obese. Similarly, it is awkward and conceptually limiting to say that a person who habitually overlooks threats to their wellbeing is very low in trait anxiety. Rather, we might say that such a person is on the low end of a trait dimension called “threat sensitivity” (Corr and McNaughton, 2008).

As defined in the Encyclopedia of Personality and Individual Differences, “Threat sensitivity refers to affective, cognitive, behavioral, and physiological responses toward threatening (likely to cause damage or danger) stimuli, information, or cues. Threat may be actual, perceived, or potential” (Denefrio and Dennis-Tiwary, 2017, p. 5503). The advantage of employing that term is that it covers the full continuum from perpetually on edge to consistently carefree, which then makes it easier to imagine a normal distribution of individuals in terms of their relative sensitivity to threat. Where a person lies along that continuum is typically assessed using self-report (Carver and White, 1994; Cloninger et al., 1994; Torrubia et al., 2008), structured interview (Brown and Barlow, 2014; First et al., 2015), or psychophysiological measures (Hyde et al., 2019). The authors of the definition of threat sensitivity above go on to state that, “High levels of threat sensitivity are associated with anxiety-, trauma-, and stressor-related disorders” (Denefrio and Dennis-Tiwary, 2017, p. 5503). Such individuals tend to experience a greater frequency and intensity of threat-related emotional responses in all of their aspects, including physiological, cognitive, motivational, affective, and behavioral (Beck et al., 1985). Even though all of the anxiety disorders share many of those characteristics, the manifest nature of the symptoms and the situations that elicit them varies across the specific diagnostic categories (Barlow, 2002; American Psychiatric Association (APA), 2013).

Most importantly, however, the present topic is about developing a greater understanding of individuals who lie at the lower, polar opposite end of the threat sensitivity continuum. These are individuals who tend to have lower scores on the self-report and structured interview measures listed above and who demonstrate much more blunted and much less frequent emotional responses to potentially threatening cues and situations.

## Mechanisms and motivations

The working hypothesis behind the empirical studies reviewed earlier is that people who lie toward the lower end of a latent continuum of threat sensitivity will: (1) either self-report or display very low levels of trait anxiety, and (2) be more likely to find or put themselves in potentially dangerous situations. But there are alternative explanations for those findings that should be considered. Maybe those men were not really low in anxiety at all. For example, could it be that the men who were particularly slow in recognizing the seriousness of their cancer symptoms were simply *denying* the possible gravity of their observations? Perhaps they were suppressing an aversive emotional reaction to their emerging symptoms at some level in an effort to calm their anxiety and postpone facing the problem. And could it be that a subset of the young Brits were merely presenting themselves as fearless in order to grow their reputations and thereby increase their schoolyard political capital? Such habits could have dire consequences. In other words, how do we know that all of these findings are not better explained by denial, stoicism, or machismo? That interpretation is bolstered by the theory that males are more likely to engage in such self-presentation tactics in order to comply with sex-based differences in societal expectations (Courtenay et al., 2002; Addis and Mahalik, 2003; Mahalik et al., 2007; Fleming and Agnew-Brune, 2015).

Given these equally plausible alternative explanations, a closer look at the underlying mechanisms of threat sensitivity could be helpful in distinguishing between the two. A greater understanding of the biological basis of the threat response would allow the investigation of more objective indicators of individual differences in threat sensitivity that could replace or complement reliance on self-report (Lukaszewski et al., 2020).

### Biological structures and mechanisms

Expanding the theoretical perspective from one half of the threat sensitivity continuum to its full breadth facilitates the mapping of behavioral data onto underlying neurobiological structures and mechanisms. Those who conduct such research tend to eschew the use of common language feeling words—such as anxiety and fear—in order not to anthropomorphize their rodent subjects nor to assume that those subjective feeling states represent a causal mediator between the presence of threat and the behavioral response (LeDoux, 2014). Instead, the focus of neurobiological research regarding threat sensitivity has been on what has variously been called the “behavioral inhibition system” (Gray, 1982) or the “defensive survival circuit” (LeDoux, 2012). The central idea that connects these formulations is that there exists a complex system of brain circuitry that serves to detect and recognize threat and then mobilize a behavioral response that will support and promote physical survival and wellbeing. As explained by LeDoux (2012), “[s]urvival circuits help organisms survive and thrive by organizing brain function. When activated [by the perception of threat], specific kinds of responses rise in priority, other activities are inhibited, the brain and body are aroused, attention is focused on relevant environmental and internal stimuli, motivational systems are engaged, learning occurs, and memories are formed” (LeDoux, 2012, p. 655).

Because defense against harm is obviously one of the very basic requirements of life (LeDoux, 2022), it stands to reason that defensive survival circuits would be hard-wired in the central nervous system. Much of that circuitry has now been traced, and it is widely understood that the amygdala plays a central role in keeping us safe from harm (Ohman, 2005; Phelps and LeDoux, 2005; Freese and Amaral, 2009; Feinstein et al., 2011). Although the amygdala may contribute to many types of self-relevant motivation and behavior (Sander et al., 2003), it is in large part a “danger detector” (Freese and Amaral, 2009). Upon receiving sensory input signaling possible threat, the amygdala forwards the alert to several other neuroanatomical structures (Davidson, 2002; Shackman et al., 2009; Salzman and Fusi, 2010; Fiddick, 2011; Clauss, 2019; Hulsman et al., 2021) and begins the elaborate orchestration of central neuromodulatory and peripheral hormonal systems that carry out the defensive response (Davis, 1992; LeDoux, 1992; Gray, 1993; LeDoux, 2012; Inman et al., 2020). The emotional “feelings” that would inform self-reports of anxiety are then derived from the conscious perception of those bodily changes (LeDoux, 2022). Self-reports of *trait* anxiety would entail the integration of memories of past threatening situations and the emotional and behavioral responses that were habitually invoked. It is most important to note, however, that the automatic response to threat and the conscious experience of anxiety depend on divergent neurocircuitry and are thus dissociable (Phelps, 2006; Hoppenbrouwers et al., 2016; LeDoux, 2020; Taschereau-Dumouchel et al., 2020; LeDoux, 2022), so that self-reports of anxiety may not always be an entirely accurate reflection of the underlying defensive response.

### Individual differences in threat sensitivity

Studies have revealed various psychophysiological markers of individual differences in threat sensitivity that would not easily be subject to conscious control nor available for self-report. Although there are no such data on the participants in the studies reviewed earlier, there are ample data on another group of individuals who also lie toward the lower tail of the threat sensitivity continuum, i.e., people who demonstrate primary psychopathic traits. Pertinent to the topic at hand, one of the cardinal features of primary psychopathy is an “absence of nervousness or psychoneurotic manifestations” (Cleckley, 1950).

In order to justify making inferences about the subjects of our review by referring to studies of primary psychopathy, two points need to be made. First, taxometric studies have shown that the latent structure that underlies primary psychopathic traits is dimensional rather than taxonic in both forensic and general populations (Marcus et al., 2004; Edens et al., 2006; Walters et al., 2007; Walters et al., 2008). Second, higher levels of primary psychopathic traits (and thus lower levels of threat sensitivity) have been assessed among (presumably) law-abiding community members and not just among sociopathic criminal offenders (Lykken, 1995; Lilienfeld et al., 2014; Benning et al., 2018; Patton et al., 2018). In other words, high levels of primary psychopathic traits alone do not qualitatively distinguish criminal offenders from others who also reside at the low end of a continuum of threat sensitivity. Thus, studies of primary psychopathic traits could shed light on the motivational and constitutional characteristics of non-forensic individuals, such as the subjects of the earlier review, who also demonstrate low levels of threat sensitivity.

The so-called “no fear” hypothesis of primary psychopathy (Lykken, 1957, 1995) has been supported many times over in experimental studies that have examined the relationship between primary psychopathic traits and automatic responsiveness to threat. Higher levels of primary psychopathic traits, whether assessed in offenders or community members, have been associated with a blunted galvanic skin response (GSR) to the threat of electric shock in a conditioning paradigm, a lesser tendency to make choices that would help them to avoid shock in a learning task, a less intense fear-potentiated startle response, muted cardiac indices of sympathetic activity in response to threat, and attenuated activity in affect-processing areas of the brain in response to emotional stimuli (Lykken, 1957; Patrick, 1994; Lykken, 1995; Seara-Cardoso and Viding, 2015; Hoppenbrouwers et al., 2016; Kozhuharova et al., 2019; Patrick, 2022). The consensus of those studies indicates a dampened processing of threat among individuals with higher levels of primary psychopathic traits, which several authors have theorized can be attributed in part to an underactive amygdala in the face of threat (Patrick, 1994; Blair, 2005; Kiehl, 2006; Jones et al., 2009; Moul et al., 2012; Seara-Cardoso and Viding, 2015; Kozhuharova et al., 2019). We might extrapolate from those findings to hypothesize that the behaviors of the subjects in the previous review were influenced by underlying neurobiological structures and mechanisms.

### Sex differences

Even if it is true that the behavioral tendencies of the subjects of the earlier review were rooted in individual differences in biologically-based responsiveness to threat, why would those tendencies be most pronounced among certain males? Related to that question are the well documented findings that males in general, compared to females in general: (1) are less likely to be diagnosed with an anxiety disorder (McLean et al., 2011; Kessler et al., 2012; Jalnapurkar et al., 2018), (2) have lower scores on measures of anxiety symptoms in community samples (Zablotsky et al., 2022), (3) exhibit less intense behavioral responses to threat in experimental paradigms (Burani and Nelson, 2020; Robinson et al., 2021), (4) are more likely to take risks (Byrnes et al., 1999), and (5) are less likely to seek medical help or to have a usual source of healthcare (National Center for Health Statistics, n.d.). Again, all these differences may be attributed to sex differences in socialization practices. It may be true that females are discouraged from engaging in risky behaviors while males are prodded by peers to engage in risky behaviors. It may also be true that females are more willing to report personal health problems because they are given greater implicit permission to do so.

On the other hand, there is also evidence showing that sex differences in certain neurobiological structures and circuits may partially account for the observation that males, compared to females, tend to be less responsive to potentially threatening situations (Butler et al., 2005; Hamann, 2005; Kilpatrick et al., 2006; Andreano et al., 2013; Clauss, 2019; Shansky, 2020; Bangasser and Cuarenta, 2021). In addition, it has been shown that sex steroid hormones have activational as well as organizational effects on those same structures and circuits, thus further contributing to sex differences in threat responsiveness (Toufexis et al., 2006; van Wingen et al., 2011; Tong et al., 2019; Singh et al., 2020; Orsini et al., 2022; Pillerova et al., 2022). There has been particular interest in testosterone, which has been shown to reduce functional connectivity between the right amygdala and the right dorsolateral prefrontal cortex (Votinov et al., 2020). Testosterone also appears to be associated with various behavioral indices of responsiveness to threat. In three related experimental studies of human females, for example, a single dose of sublingual testosterone resulted in a reduced GSR to viewing threatening pictures (Hermans et al., 2007), a reduction in magnitude of the fear-potentiated startle reflex (Hermans et al., 2006), and longer times to recognize angry—but not fearful or disgusted—faces (van Honk and Schutter, 2007), all indicating a dampened sensitivity to threat. Endogenous testosterone, when considered as a marker of individual differences in dispositional tendencies (Sellers et al., 2007; Newman and Josephs, 2009), has also been associated with individual differences in sensitivity to threat. In a study of undergraduate males, for instance, those with higher levels of basal testosterone gave lower estimates of the seriousness of a number of medical conditions as well as of a (fictitious) condition with which they had just been “diagnosed” (Ristvedt et al., 2012). In other words, the high testosterone males were in essence downplaying the threat of those medical conditions.

Thus the studies presented so far in this section open the possibility that the men in the previous review of empirical studies were acting, at least in part, in accordance with their biological constitution and not strictly in alignment with a desire to conform to societal expectations (Courtenay et al., 2002; Addis and Mahalik, 2003; Fleming and Agnew-Brune, 2015; Weber et al., 2019).

Differences between males and females in how they perceive and respond to potentially threatening situations could also involve sex differences in the decision-making process that results in an estimate of the dangerousness of the situation. The hypothesis to be entertained here is that males are more likely than females to succumb to the “affect heuristic” (Slovic et al., 2002) when judging the threat value of a certain situation or stimulus (Finucane et al., 2000; Loewenstein et al., 2001; Slovic et al., 2005; Slovic and Peters, 2006). The term affect heuristic refers to the observation that the “goodness” or “badness” of one’s affective state can have an influence on his or her assessments of the “goodness” or “badness” of a target stimulus (Slovic et al., 2002). Notably, there are differences among individuals in their reliance on such heuristics (Berthet and de Gardelle, 2023).

If the affect heuristic is indeed at play in the present context, we might expect that a male who is low in threat sensitivity will be more likely to say to himself, “If I do not feel threatened, then I must not be in danger.” That same person might then continue to neglect developing signs of rectal cancer or might then proceed with behaviors that could lead to a serious or even fatal accident. Going further, we might expect that a male who is *high* in threat sensitivity will be more likely to say to himself, “If I feel threatened, then I must be in danger.” That person would then be much quicker to recognize the seriousness of his emerging cancer symptoms and much less likely to engage in activities that could result in accidental injury or death. Furthermore, if females tend not to succumb to the affect heuristic, we would expect there to be no association between their levels of threat sensitivity and their responses to potentially threatening situations. This hypothesis was derived from a closer examination of the data from all three of the empirical studies reviewed earlier that included subjects of both sexes. In the first study of the neglect of serious physical symptoms, it was found that the males who had the lowest scores on the TCI-HA were the ones who took the longest to recognize the seriousness of their symptoms compared to all the other subgroups (see Figure 1). Conversely, it was found in that study that the males who had the *highest* scores on the TCI-HA were the ones who were the *quickest* to recognize the seriousness of their symptoms (Mdn = 2.5 weeks) compared to all the other subgroups. In contrast, no clear association was found among the females between their TCI-HA scores and their symptom appraisal times (Mdn = 9.0 weeks for low TCI-HA scorers and 12.0 weeks for high TCI-HA scorers). In the second study of symptom appraisal, the males who had the highest scores on the BIS were again the quickest to recognize the seriousness of their symptoms (Mdn = 2.0 weeks) compared to all the other subgroups. The third place where a similar pattern was found was in the study of accidental injuries. As can be seen in Table 2, there was a significant association between teacher’s assessments of their male students’ anxiety levels and the likelihood that those students would later sustain serious accidental injuries. Males who were judged to be lower in anxiety were much more likely to sustain such injuries than males who were judged to be higher in anxiety (OR = 1.2). On the other hand, there was no association between anxiety level and likelihood of accidental injury among the females (OR = 1.0). These findings from these three studies all lend support to the hypothesis that males are more likely than females to succumb to the affect heuristic when judging the threat value of a certain situation or stimulus. If this hypothesis is further supported in future research, it may have implications for the study of sex differences in the neurobiological structures and functions that underlie the processing of threat.

## Discussion

In this paper I have presented evidence of a personality trait characteristic, i.e., low threat sensitivity, that is associated with a tendency to engage in behaviors that could jeopardize one’s physical wellbeing. That association seems to be especially true among males. That relationship was illustrated in the initial review of studies that reported on an array of outcomes including the decision to take part in high-risk climbing expeditions, the repeated dismissal of cancer warning signs, going without a usual source of medical care, and getting into situations that could lead to accidental injury or even death. The general hypothesis that was derived from that review is that when attention to everyday threats is chronically muted by way of a dispositional trait, the likelihood of proceeding down some dangerous path is increased.

I have also presented evidence, admittedly circumstantial, suggesting that these behaviors may be partly rooted in an innately underactive, biologically-based defensive survival circuit (LeDoux, 2012). Some of that evidence also supports speculation about why these behaviors may be more often seen among certain males given sex (and inter-individual) differences in brain structures and hormonal profiles. The essence of this claim is that the greater prevalence of neglect of personal safety and health-related matters among some males might be influenced by biologically-based motivational differences, perhaps in a complementary relationship with an acquired motivation to conform to social norms of masculinity that model neglect of personal health and safety (Courtenay et al., 2002; Addis and Mahalik, 2003; Fleming and Agnew-Brune, 2015; Weber et al., 2019).

However, low sensitivity to threat would probably not in itself motivate imprudent behaviors, but might simply set the stage for them. One person who is very low in threat sensitivity might be eager to join Teddy on his trip while another would have no desire to go along. Thus, it is likely a combination of motivational tendencies that lands certain people in harm’s way. As an illustration, recall that the men who had reached the summit of Mount Everest in the Feldman et al. study (2013), compared to those who had not, had not only lower scores on the anxiety measure but also had higher scores on the BAS Reward Responsiveness subscale (Carver and White, 1994). Thus, a muted motivation to protect oneself from harm combined with an enhanced motivation to pursue rewarding experiences propelled those climbers higher into risky territory.

This dynamic is one example of what has been dubbed “motivational imbalance,” meaning that one basic motivation (i.e., defense against harm) is decreased while another, often opposing, motivation (i.e., pursuit of reward) is increased (Kruglanski et al., 2021). According to Kruglanski and colleagues, an imbalance in motivations can lead to extreme behaviors such as mountain climbing in the previous example. Furthermore, that imbalance in motivations is likely associated with a similar dynamic in underlying neural circuitry. That is, when two circuits support opposing motivations, the suppression of one circuit allows the activation of the other (LeDoux, 2015). Take the even more extreme example of people who embark on long and potentially treacherous treks, such as that undertaken by Roosevelt and his crew. Qualitative studies of these “extended-period expeditionary adventurers,” show that they are strongly motivated to bring about “the expansion of their geographical, physical and psychological worlds” (Reid and Kampman, 2020). It may be the combination of an underactive defensive survival circuit and a hyperactive “expansion circuit” that compels these people to do what they do. In that regard, it has been pointed out that constitutionally-based low threat sensitivity can underlie various behavioral manifestations of phenotypical boldness, which has been characterized by “a capacity to remain calm and focused in situations involving pressure or threat, an ability to recover quickly from stressful events, high self-assurance and social efficacy, and a tolerance of unfamiliarity and danger” (Patrick et al., 2009, p. 926). Even though dispositionally low sensitivity to threat raises the likelihood of unfortunate outcomes, it also sets the stage for great achievements for the individual and the species, which furthermore suggests the adaptive potential of this particular personality trait (Lukaszewski et al., 2020).

Parenthetically, there is some evidence that hormonal moderators play a role in both sides of this motivational imbalance. It was noted earlier that higher levels of testosterone—whether exogenous or endogenous—are associated with a dampened sensitivity to threat. On the other side of the motivational imbalance, hormonal profiles may also play a role in various manifestations of reward (vs. threat) sensitivity (Gray, 1994). For example, studies have consistently shown that males demonstrate a greater propensity than females toward sensation-seeking (Cross et al., 2013), which has been associated with many forms of risky behavior (Zuckerman, 2007). In that regard, tendencies toward sensation-seeking may have been at play in the previously cited studies of mountain climbing and accidental injuries and death. Pertinent to the present discussion, greater tendencies toward sensation-seeking have been associated with higher levels of endogenous testosterone and lower levels of endogenous estradiol (Harden et al., 2018). In addition, higher levels of testosterone, particularly in combination with low levels of cortisol, have been associated with a variety of behavioral operationalizations of boldness, such as financial risk-taking (Herbert, 2018), achievement in the corporate world (Sherman et al., 2016), and dominance striving in general (Mehta and Josephs, 2010; Stanton and Schultheiss, 2011; Hamilton et al., 2015; Mehta and Prasad, 2015). It is intriguing to speculate that certain configurations of neurobiological structures and hormonal profiles are associated with a heightened motivation to expand one’s personal, societal, and geographic footprint.

However, much more common scenarios that involve associations between low threat sensitivity and the careless temptation of fate are illustrated in the earlier review of studies involving medical patients and community members, most of whom are likely neither super achievers nor psychopaths. Even though the focus of those studies was on low threat sensitivity, there may still have been an imbalance in motivations that increased the chance of bad things happening. When a person’s guard is habitually down, other daily activities are given greater attention and competing motivations are allowed freer reign. For example, rather than giving adequate attention to the emergence of unusual bodily changes, the afflicted person might be mentally consumed with plotting out strategies for personal career advancement. And rather than taking the time to arrange for ongoing medical care, someone else could be preoccupied with maintaining an active social life.

### Future directions

In conclusion, many of the claims made in this paper might be seen as tenuous leaps between previously unconnected areas of research. All of those areas of research were included with the intention to support the central claim of this paper, which is that low sensitivity to threat can have unfortunate consequences, particularly for men. However, that relationship is surely complicated in real life by a number of personal, social and environmental moderating factors that were not investigated here. It is hoped that any missing pieces or gaps in logic that are detected by the reader will be developed into testable questions for future research. Here are a few possibilities. First, are there more examples of imprudent behaviors that are associated with a sex-by-threat sensitivity interaction? The empirical studies described earlier cover a very broad range of threat sensitivity measurement strategies and types of imprudent behaviors. The plausibility of the central claim of this paper would be enhanced by studies—perhaps experimental—that would provide greater convergence on the constructs of interest. Second, are there moderators that could explain any differences between behaviors that replicate the sex-by-threat sensitivity interaction and those that do not? Consideration should be given to contextual factors that could impact decision-making in the face of threat, such as social influences or environmental constraints. Consideration should also be given to intraindividual variability in how a person responds to potential threats. That is, one can imagine a person who is indifferent in the face of physical threat but who is also hypervigilant in situations where there is the possibility of social threats, such as the risk of ostracism or embarrassment. Lastly, are there ways to investigate the interplay between innate and learned characteristics of individuals who demonstrate a habitual tendency to put their personal wellbeing at risk?

## Data Availability

The original contributions presented in the study are included in the article/supplementary material, further inquiries can be directed to the corresponding author.
